# Nondestructive determination of ash content in wheat flour via terahertz time-domain spectroscopy

**DOI:** 10.3389/fpls.2026.1843188

**Published:** 2026-05-19

**Authors:** Xin Wu, Guanglin Li, Yin Shen, Weixin Wu, Zhengdong Li

**Affiliations:** 1 College of Engineering and Technology, Southwest University, Chongqing, China; 2 School of Electronics and Internet of Things, Chongqing Polytechnic University of Electronic Technology, Chongqing, China; 3 College of Artificial Intelligence Medicine, Chongqing Medical University, Chongqing, China; 4 Mechanical Measurement and Testing Research Center, Academy of Metrology and Quality Inspection, Chongqing, China; 5 College of Intelligent Transportation, Chongqing Vocational College of Public Transportation, Chongqing, China

**Keywords:** ash content, competitive adaptive reweighted sampling (CARS), partial least squares regression (PLSR), terahertz time-domain spectroscopy (THz-TDS), wheat flour

## Abstract

As a global dietary staple, wheat flour is a primary commodity in the milling industry, where ash content serves as a critical indicator of flour purity, grade, and milling efficiency. Traditional incineration methods are time-consuming and labor-intensive. This study explores the potential of employing Terahertz time-domain spectroscopy (THz-TDS) coupled with chemometric algorithms for the rapid, nondestructive quantification of ash content in wheat flour. Based on three distinct wheat cultivars (*Nongmai 126*, *Zhongyou 206*, and *Changhan 58*), a total of 183 samples were analyzed using a fiber-optic coupled THz-TDS system. The raw time-domain signals were transformed into frequency-domain signals, absorption coefficients, transmittance, and refractive index for comparative evaluation. Among these optical parameters, the absorption coefficient exhibited the most robust correlation with ash content, attributed to the distinct spectral fingerprints of inorganic mineral constituents. Savitzky–Golay (SG) smoothing was identified as the optimal preprocessing technique to attenuate stochastic noise. Competitive adaptive reweighted sampling (CARS) was subsequently implemented to screen 31 characteristic wavelengths closely associated with ash components. To optimize predictive performance, four regression frameworks-Partial Least Squares Regression (PLSR), Multiple Linear Regression (MLR), Principal Component Regression (PCR), and Support Vector Regression (SVR)-were constructed and rigorously compared. The optimized SG-CARS-PLSR model achieved superior predictive accuracy, yielding a correlation coefficient of prediction (Rp) of 0.976, a root mean square error of prediction (RMSEP) of 0.011%, the Ratio of Performance to Deviation (RPD) of 4.804, and the Bias of 0.004%. These findings suggest that THz-TDS, integrated with CARS feature selection and PLSR modeling, provides a rapid, reliable, and nondestructive methodology for the quantitative analysis of ash content in wheat flour.

## 1 Introduction

Wheat flour, as a fundamental powdered product derived from wheat kernels, represents the most critical output in the wheat milling industry, utilized extensively in baking, confectionery production, pasta manufacturing, and food concentrate processing, including breads, cakes, biscuits, and noodles (Czaja et al., 2020; Zhang et al., 2022). Its global significance is underscored by its role as a major consumable in daily diets, providing essential nutrients such as carbohydrates, proteins, and minerals (Li et al., 2023).

The quality assessment of wheat flour hinges on key parameters, among which ash content stands as a pivotal indicator (Gordon and Nancy, 2009; Cardoso et al., 2019). Ash, comprising mineral elements like calcium, magnesium, phosphorus, potassium, iron, zinc, and copper (Kulkarni et al., 2006; Vieno et al., 2009), directly reflects the milling degree and extraction rate of flour (Dong and Sun, 2013; Czaja et al., 2020). The whole wheat grain contains 1.17-2.96% of the mineral constituents (Obert et al., 2004). It functions as a crucial metric for evaluating flour purity, processing accuracy, and suitability for specific applications (Li et al., 2023). Higher ash levels typically indicate greater extraction rates and reduced purification, correlating with increased presence of bran particles and endosperm adjacent to the bran. While elevated ash content enhances nutritional value by boosting dietary fiber, vitamins, and non-gluten proteins (Hemery et al., 2011), it concurrently diminishes technical quality-manifesting as darker color, heightened enzymatic activity, and weakened dough structure due to interference from dietary fiber and non-gluten proteins (Gordon and Nancy, 2009; Bucsella et al., 2016). Consequently, ash content remains an indispensable parameter for comprehensive flour quality evaluation, influencing both nutritional labeling and processing performance.

The determination of ash content in wheat flour conventionally relies on standardized methods, primarily based on high-temperature incineration as outlined by protocols such as AACC 8–01 and ICC 104-1 (ICC, 1994; AACC, 2002). These methods involve burning the sample at specific temperatures, typically 550 °C for soft wheat flour and 575 - 590 °C for hard wheat flour, until a constant weight of light gray ash is achieved, a process that is inherently destructive and typically requires 5 to 7 hours (Bilge et al., 2016; Sezer et al., 2017). While these conventional approaches, which may also include supplementary physical or chemical techniques like weighing residues or configuring reagents to measure precipitates, are valued for their good precision, accuracy, specificity, and sensitivity, they are characterized by significant practical drawbacks. The procedures are notably laborious, time-consuming, technically challenging, and demand considerable manual work (Dong and Sun, 2013; Czaja et al., 2020; Zhang et al., 2022). Operational complexity and lengthy processing times preclude their use in rapid batch screening and effective real-time quality assurance within industrial settings.

While conventional incineration methods remain the industry standard, their operational complexity and length preclude effective real-time quality assurance. Consequently, research has shifted toward rapid, nondestructive instrumental techniques. Near-infrared (NIR) spectroscopy has emerged as a dominant tool, with recent reviews highlighting its evolution toward handheld and online systems for simultaneous chemical and safety analysis (Zhang et al., 2022). Furthermore, the integration of lightweight convolutional neural networks (CNNs) and attention modules has significantly enhanced the precision of NIR-based real-time monitoring of moisture, protein, and ash contents (Yang et al., 2025). Beyond NIR, hyperspectral imaging (HSI) coupled with data fusion techniques has demonstrated superior performance in characterizing ash distribution across different wheat varieties, offering a more granular assessment than single-point measurements (Li et al., 2023). Raman spectroscopy has also seen renewed interest; recent advancements in laser excitation and detector sensitivity now allow for molecular-level insights into starch and gluten structures, facilitating in-line monitoring of flour functionality in bakery applications (Van Der Vennet et al., 2025). Although techniques such as laser-induced breakdown spectroscopy (LIBS) and FT-IR continue to be explored for their high sensitivity to mineral constituents, the implementation of terahertz time-domain spectroscopy (THz-TDS) offers a unique advantage by probing the low-frequency vibrational modes of inorganic crystals and hydrogen-bonded networks, which are directly related to ash-forming minerals.

Terahertz time-domain spectroscopy (THz-TDS) has emerged as a powerful nondestructive detection tool in the agricultural and food industries, capable of identifying biological molecules through their unique spectral fingerprints and evaluating optical properties without the need for chemical reagents or sample pretreatment (K. Mathanker et al., 2013; Qin et al., 2013; Jiang et al., 2014; Ge et al., 2016; Liu et al., 2018). In recent years, the integration of THz technology with advanced chemometric algorithms has significantly advanced food safety and quality analysis, demonstrating high efficacy in applications such as discriminating transgenic edible oils using Weighted Linear Discriminant Analysis (WLDA) (Liu et al., 2019) and detecting insect foreign bodies in tea with prediction accuracies reaching 100% via adaptive iteratively reweighted penalized least squares (AirPLS) correction (Sun et al., 2021). Within the specific domain of wheat flour analysis, THz spectroscopy has proven particularly valuable; for instance, combined with back propagation neural networks (BPNN), it achieved high accuracy in determining low-concentration ternary pesticide mixtures, the Rp were 0.9913, 0.9948, 0.9923, and corresponding RMSE were 0.0211%, 0.0176%, 0.0191% (Ma et al., 2022), and coupled with Least Squares Support Vector Machine (LS-SVM), it effectively quantified benzoic acid additives in wheat flour, with a Rp of 0.994 and RMSEP of 0.12% (Sun et al., 2019). Regarding ash detection, Li et al (Li et al., 2023). recently explored the potential of fusing hyperspectral imaging with THz technology, utilizing a Hierarchical Extreme Learning Machine (H-ELM) model that achieved impressive results (r^2^ = 0.989 and RMSEP = 0.015%). Despite these promising developments and the general consensus that spectroscopy offers a rapid and effective approach to quality assessment, current THz detection models specifically for ash content in wheat flour still require enhancement due to limitations in detection accuracy.

The objective of this study was to employ THz-TDS for the nondestructive determination of ash content in wheat flour, addressing the existing need for enhanced model precision and reliability. The research procedures of this study are as follows: (1) A THz-TDS data acquisition system for wheat flour was set up to collect THz-TDS data; (2) The standard reference values of ash content in wheat flour were determined through high-temperature incineration; (3) The THz-TDS data were transformed into frequency-domain, absorption coefficient, transmittance, and refractive index, and Partial Least Squares Regression (PLSR) calibration models for ash determination were established respectively; (4) The optimal calibration model was obtained by exploring different combinations of absorption data preprocessing methods (SG, 1D, 2D, SNV and MSC); (5) SPA, CARS, GA, and BOSS were utilized to select characteristic spectra for establishing the PLSR model for measurement for ash content in wheat flour; (6) The performance of the optimal calibration model, constructed on characteristic spectra and modeling algorithms (PLSR, MLR, PCR, and SVR), was evaluated using independent prediction set data.

## 2 Materials and methods

### 2.1 Sample preparation and ash content measurement

**Figure 1:**
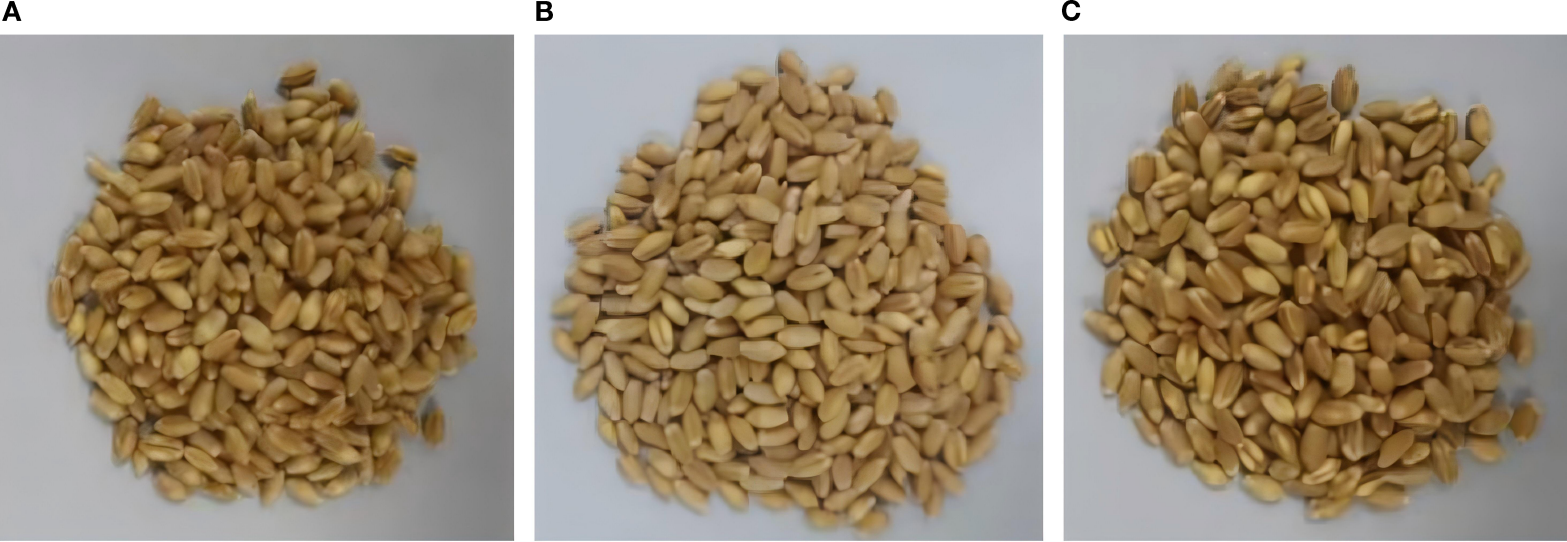
Samples of different varieties of wheat. **(A)** Nongmai 126, **(B)** Zhongyou 206, and **(C)** Changhan 58.

**Figure 2:**
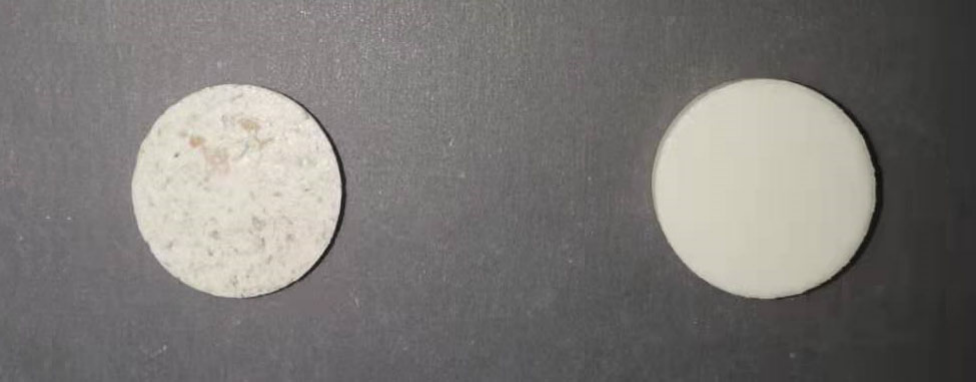
Wheat flour-PE composite tablets (left) used for spectral acquisition and pure polyethylene (PE) tablets (right) used as reference blanks.

The experimental samples were sourced from the Chongqing Academy of Agricultural Sciences. The wheat varieties comprised *Nongmai 126, Zhongyou 206*, and *Changhan 58* (presented in **Figure 1**), which are extensively cultivated in the southwestern region of China. A total of 183 samples were gathered. The wheat samples were milled and subsequently dried in an electric thermostatic drying oven to decrease the moisture content. The milled and dried samples were sifted through a 200-mesh sieve (75μm aperture) to produce wheat flour. The ash content was determined in accordance with the GB5009.4–2016 standard, which involves burning equal weights of the sample in a chamber furnace at 550 °C ± 25 °C until no carbon particles were present in the wheat flour. After cooling the burnt samples in a dryer, the ash content was weighed. In the experiment, 100 mg of wheat flour was weighed from each sample and mixed with 20 mg of polyethylene (PE) powder (Sigma-Aldrich, USA) to achieve a 5:1 (w/w) ratio. This specific proportion serves as a binder to ensure the mechanical stability of the resulting pellet and to minimize potential scattering effects during terahertz transmission. The mixture was thoroughly homogenized in an agate mortar for 5 minutes. Subsequently, the homogenized mixture was subjected to a pressure of 3 tons for 2 minutes using a manual hydraulic press, forming a thin disc with a thickness of 1.2 ± 0.5 mm and a diameter of 13 mm (as shown in **Figure 2**).

### 2.2 THz-TDS acquisition

**Figure 3:**
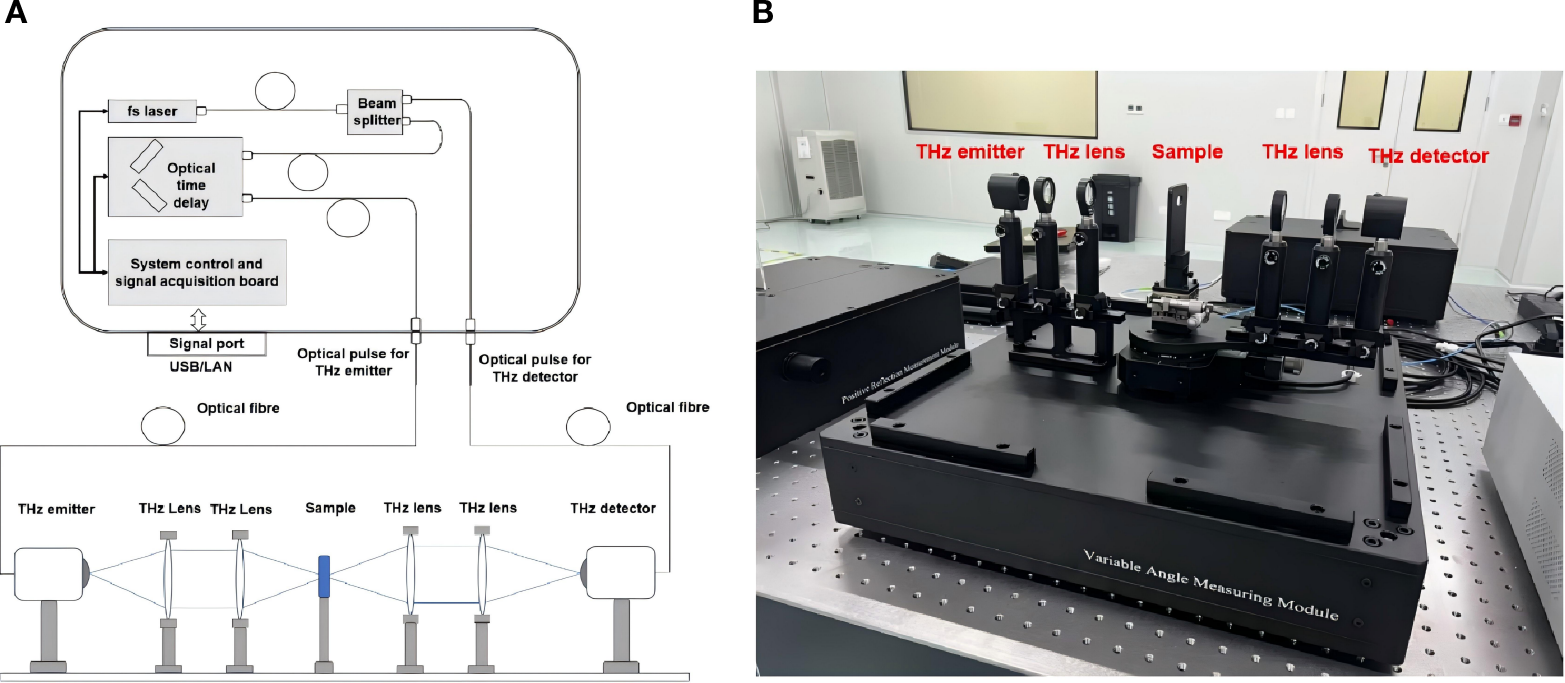
The THz-TDS system for spectral measurement. **(A)** structure diagram of system. **(B)** field assembly diagram of system.

The fiber optic coupled THz-TDS system used in this paper is purchased from Chongqing Terasmart Co. Ltd, which features a streamlined structure designed for efficient terahertz signal generation, transmission, and detection, as illustrated in **Figure 3**. The system has a spectral range of 0.1–4.5 THz, a spectral resolution of less than 4 GHz, and a dynamic range of 70 dB. It typically includes a femtosecond laser as the light source (with a center wavelength of 1560 ± 10 nm and an average power of 80 mW (typical value 100 mW)), a fiber coupling module for precise light delivery, a THz generation and detection unit using photoconductive antennas, a delay line for time-domain measurements, and a sample holder for positioning the specimen. The system also integrates a high-speed digitizer and data processing unit to capture and analyze the THz signals.

To measure the samples, the THz beam generated by the system is directed through the sample placed in the holder. By using an air purged container, the experiment was carried out at room temperature in dry air with a relative humidity of 0%. Three measurements were recorded for each sample to avoid the random errors. The transmitted THz signal is then captured by the detector. By comparing the amplitude and phase of the THz signal before and after passing through the sample, the transmittance can be calculated. This process involves recording the time-domain waveform, performing a Fourier transform to obtain the frequency-domain spectrum, and analyzing the changes in the THz signal to determine the sample’s optical properties and transmittance characteristics.

### 2.3 Dataset partitioning

To develop and validate the quantitative regression models, the 183 wheat flour samples were partitioned into a calibration set and a prediction set. A stratified Kennard-Stone (KS) algorithm was applied to the dataset, using the three wheat cultivars as strata to ensure a balanced representation of biological diversity in both subsets. Following a ratio of approximately 3:1, 137 samples were assigned to the calibration set for model training and internal 10-fold cross-validation, while the remaining 46 samples were reserved as an independent prediction set. Crucially, the prediction set was held out before any spectral preprocessing or characteristic wavelength selection to prevent data leakage and provide a rigorous assessment of the models’ generalization performance.

### 2.4 THz spectral signals preprocessing method

In the present study, the raw THz spectral signals acquired from the measurements were preprocessed utilizing Unscrambler X v. 10.4 software (Camo Analytics, Oslo, Norway). A series of preprocessing operations were executed, including baseline correction, scattering correction, spectral smoothing, and scale normalization (Gautam et al., 2015; Li et al., 2018). These operations were aimed at eliminating non-target interference factors in the raw spectral signals, thereby enhancing the reliability and interpretability of subsequent modeling analysis. Specifically, Savitzky–Golay (SG) smoothing was employed to suppress random noise in the spectral signals and enhance the signal-to-noise ratio of the sample spectra. The first derivative (D1) and second derivative (D2) transformations were utilized to eliminate the adverse effects of instrumental background interference and baseline drift on the spectral signals, respectively. Additionally, multiplicative scatter correction (MSC) and standard normal variate (SNV) transformation were implemented as two typical scattering correction methods to minimize the spectral deviations caused by light scattering effects. These effects arise from factors such as sample particle size inhomogeneity and uneven sample packing in the detection cell.

### 2.5 Characteristic THz spectral signals extracting method

In order to enhance the predictive performance of calibration models for wheat flour ash content detection, characteristic spectral variables were extracting on the preprocessed THz spectral signals. This process was intended to screen spectral variables with a strong linear or non-linear correlation to ash content, while eliminating redundant, irrelevant, and noisy spectral information (Sun et al., 2020). Which reduces the dimensionality of the raw spectral data, mitigates the multicollinearity among spectral variables, and ultimately enhances the computational efficiency and generalization ability of subsequent quantitative calibration models. The Successive Projections Algorithm (SPA) effectively reduces multicollinearity among variables through stepwise projection optimization (Li et al., 2014a; Liu et al., 2017; Tao et al., 2019). However, it may select less useful variables or even interference variables. Competitive Adaptive Reweighted Sampling (CARS) iteratively eliminates variables with small absolute regression coefficients based on adaptive reweighted sampling and exponential function decay (Li et al., 2009; Jiang et al., 2015; Hu et al., 2018; Bai et al., 2019). Nevertheless, this forced elimination mechanism risks the loss of potentially useful low-contribution variables. The genetic algorithm (GA) initializes a population of random variables and then iteratively performs selection, crossover, and mutation operations on individual variables based on the fitness function to identify the optimal feature combination (Du et al., 2019). However, it carries a high risk of overfitting, which can decrease computational efficiency and undermine the predictive stability of the established models. Bootstrapping soft shrinkage (BOSS) integrates weighted bootstrap sampling (WBS) with soft shrinkage of regression coefficients, which adaptively selects characteristic wavelengths by evaluating the absolute value of regression coefficients in the entire variable space, achieving adaptive dimensionality reduction without forced elimination of low-weight variables (Deng et al., 2016; Yan et al., 2019). In this study, SPA, CARS, GA and BOSS were comprehensively employed to extract characteristic spectral variables that exhibit the most significant response to variations in wheat flour ash content. The extracted characteristic spectral variables were then validated for their effectiveness by comparing the modeling results of the full-spectrum data and the characteristic spectral data, ensuring that the selected characteristic data could accurately reflect the intrinsic correlation between THz spectral information and wheat flour ash content.

### 2.6 Machine learning modeling

In this study, we employed four classical machine learning regression algorithms-Partial Least Squares Regression (PLSR), Multiple Linear Regression (MLR), Principal Component Regression (PCR), and Support Vector Regression (SVR)-to develop quantitative estimation models for determining ash content in wheat flour. A comprehensive comparative analysis of the performance of these four algorithms was conducted to identify the optimal regression model for detecting ash content based on THz spectral signals. To develop robust quantitative models for ash content, four established regression algorithms were implemented using MATLAB 2016b software (MathWorks, USA).

PLSR was employed as the primary linear modeling technique due to its ability to handle high-dimensional, collinear THz spectral data by extracting latent variables (LVs) (Haaland and Thomas, 1988). In this study, the optimal number of LVs was determined by minimizing the root mean square error of cross-validation (RMSECV) to prevent overfitting. MLR was applied to the characteristic wavelengths identified by feature selection algorithms (CARS, SPA, etc.) to establish a transparent linear relationship between spectral intensity and ash content (Li et al., 2014b, Liu et al., 2017; Wang et al., 2017). To ensure model stability, MLR was strictly restricted to low-dimensional feature sets to avoid the “curse of dimensionality” and multicollinearity inherent in full-spectrum THz data. PCR was utilized to regress the ash content against orthogonal principal components (PCs) derived from the THz absorption spectra. This approach facilitated dimensionality reduction while retaining the maximum variance of the original signal (Xiaobo et al., 2010; Ge et al., 2016; Huang and Yu, 2019). The number of PCs was optimized through internal cross-validation based on the cumulative variance contribution rate, with a threshold of 95%. To account for potential nonlinearities in the THz response, SVR was implemented using a Radial Basis Function (RBF) kernel (Zhao et al., 2024). The model hyperparameters, specifically the penalty coefficient (C) and the kernel function parameter (γ), were optimized via a grid-search (GS) procedure with 10-fold cross-validation to maximize predictive accuracy. In this study, the grid search range for hyperparameters was set as C ∈ [2^-5^, 2^10^] and γ ∈ [2^-10^, 2^5^], with the RMSECV as the objective function to select the optimal parameter combination for each kernel function.

### 2.7 Model evaluation

The performance of the established terahertz (THz) spectral calibration models for ash content determination was comprehensively evaluated using six quantitative indicators widely adopted in spectral chemometrics. The correlation coefficient of the calibration set (Rc), the root mean square error of cross-validation (RMSECV), the correlation coefficient of the prediction set (Rp), and the root mean square error of prediction (RMSEP) were used as basic metrics (Liu and Guo, 2017; Tian et al., 2018). Furthermore, Ratio of Performance to Deviation (RPD) and Bias were introduced to evaluate the practical prediction capability and systematic error, respectively.

For the calibration set, where n represents the number of samples, the Rc is calculated as follows (**Equation 1**):

(1) $$R_{c}=\sqrt{1-\frac{\sum_{i=1}^{n}{\left(y_{i,a}-y_{i,p}\right)}^{2}}{\sum_{i=1}^{n}{\left(y_{i,a}-y_{i,cm}\right)}^{2}}}$$

The RMSECV is given by (**Equation 2**):

(2) $$RMSECV=\sqrt{\frac{\sum_{i=1}^{n}{\left(y_{i,a}-y_{i,p}\right)}^{2}}{n}}$$

Here, *y_i_*,*_a_* is the standard reference value for the i-th sample, *y_i_*,*_p_* is the predicted value for the i-th sample, and *y_i_*,*_cm_* is the mean value of the standard references for all samples.

Similarly, for the prediction set, where m denotes the number of samples, the Rp is calculated as (**Equation 3**):

(3) $$R_{p}=\sqrt{1-\frac{\sum_{j=1}^{m}{\left(y_{j,a}-y_{j,p}\right)}^{2}}{\sum_{j=1}^{m}{\left(y_{j,a}-y_{j,pm}\right)}^{2}}}$$

And the RMSEP (**Equation 4**), RPD (**Equation 5**), Bias (**Equation 6**) are given by:

(4) $$RMSEP=\sqrt{\frac{\sum_{j=1}^{m}{\left(y_{j,a}-y_{j,p}\right)}^{2}}{m}}$$

(5) $$RPD=\frac{SD_{pred}}{RMSEP}$$

(6) $$Bias=\frac{1}{m}\sum_{j=1}^{m}\left(y_{j,a}-y_{j,p}\right)$$

In these equations, *y_j_*,*_a_* is the standard reference value for the j-th sample, *y_j_*,*_p_* is the predicted value for the j-th sample, *y_j_*,*_pm_* is the mean value of the standard references for all samples, *SD_pred_* is the standard deviation of the standard reference values in the prediction set.

The criteria for evaluating RPD are as follows: an RPD value below 1.5 indicates the model is not usable, values ranging from 1.5 to less than 2.0 suggest rough prediction capability, those from 2.0 to less than 2.5 mean the model is quantitatively applicable, and an RPD of 2.5 or higher signifies excellent model performance (Chang et al., 2001). Meanwhile, a Bias value close to 0 implies no significant systematic overestimation or underestimation. Higher Rc and Rp (closer to 1), lower RMSECV and RMSEP, higher RPD, and Bias near 0 represent better model accuracy, robustness, and practicality.

## 3 Results and discussion

### 3.1 Reference measurements of ash content

The descriptive statistics for the ash content are summarized in **Table 1**. The total sample set (n=183) exhibited an ash content range of 0.36% to 0.71%, with a mean of 0.5233% and a SD of 0.0684. Utilizing the Kennard stone (KS) algorithm, the samples were divided into a calibration set (n=137) and a prediction set (n=46), ensuring the representativeness of the sample distribution. The calibration set exhibits the highest mean (0.5306%) and largest standard deviation (SD = 0.0714), the prediction set shows a lower mean (0.5014%) and smaller SD (0.0536). The calibration set covers the entire range of the total set, ensuring the model is trained on the full spectrum of available variability. Statistical analysis via an independent-samples t-test revealed a significant difference between the mean ash content of the calibration and prediction sets (p = 0.012). This distributional shift is a common byproduct of the Kennard-Stone algorithm, which prioritizes uniform coverage of the spectral space over matching the concentration means. However, the calibration set’s range (0.36%- 0.71%) completely brackets the prediction set (0.3849%-0.6684%). This ensures that the model operates in an interpolation mode during validation, which is the primary requirement for predictive reliability in chemometrics.

**Table 1:** Descriptive statistics of ash content (%) in wheat flour samples for the total, calibration and prediction sets.

Dataset	Number of samples	Minimum (%)	Maximum (%)	Mean (%)	Standard deviation (SD)
Total sample Set	183	0.3600	0.7100	0.5233	0.0684
Calibration Set	137	0.3600	0.7100	0.5306	0.0714
Prediction Set	46	0.3849	0.6684	0.5014	0.0536

**Figure 4:**
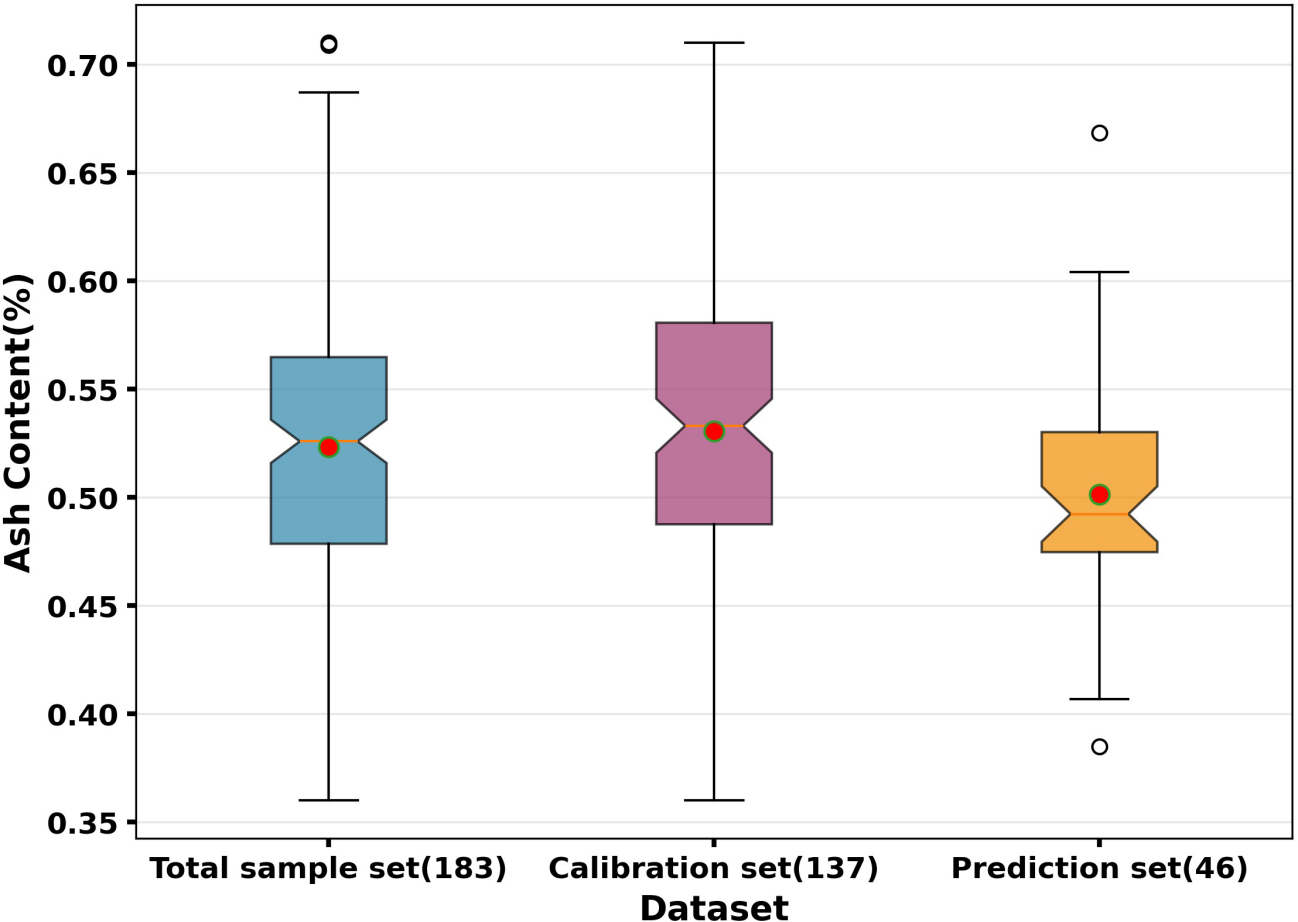
Box plot analysis of ash content distribution. Points located beyond the whiskers (1.5 × IQR rule from the median) denote mild outliers which were retained to ensure the model captures the full range of milling variability.

The distribution of these values across the calibration and prediction sets is visually compared in the box plot in **Figure 4**, outliers were identified using the Tukey method (1.5 × IQR rule) in the box plot analysis. Several ‘mild outliers’ were identified at the high end of the ash content spectrum (above 0.67%). These samples were purposely retained in both the calibration and prediction sets. These values represent flours with a higher inclusion of bran particles, reflecting the natural variability found in commercial milling. By retaining these samples, the model is trained to recognize the full breadth of the ash content range, thereby enhancing its robustness for practical industrial applications, despite the potential for slightly higher RMSEP values.

### 3.2 Analysis of THz-TDS signals

**Figure 5:**
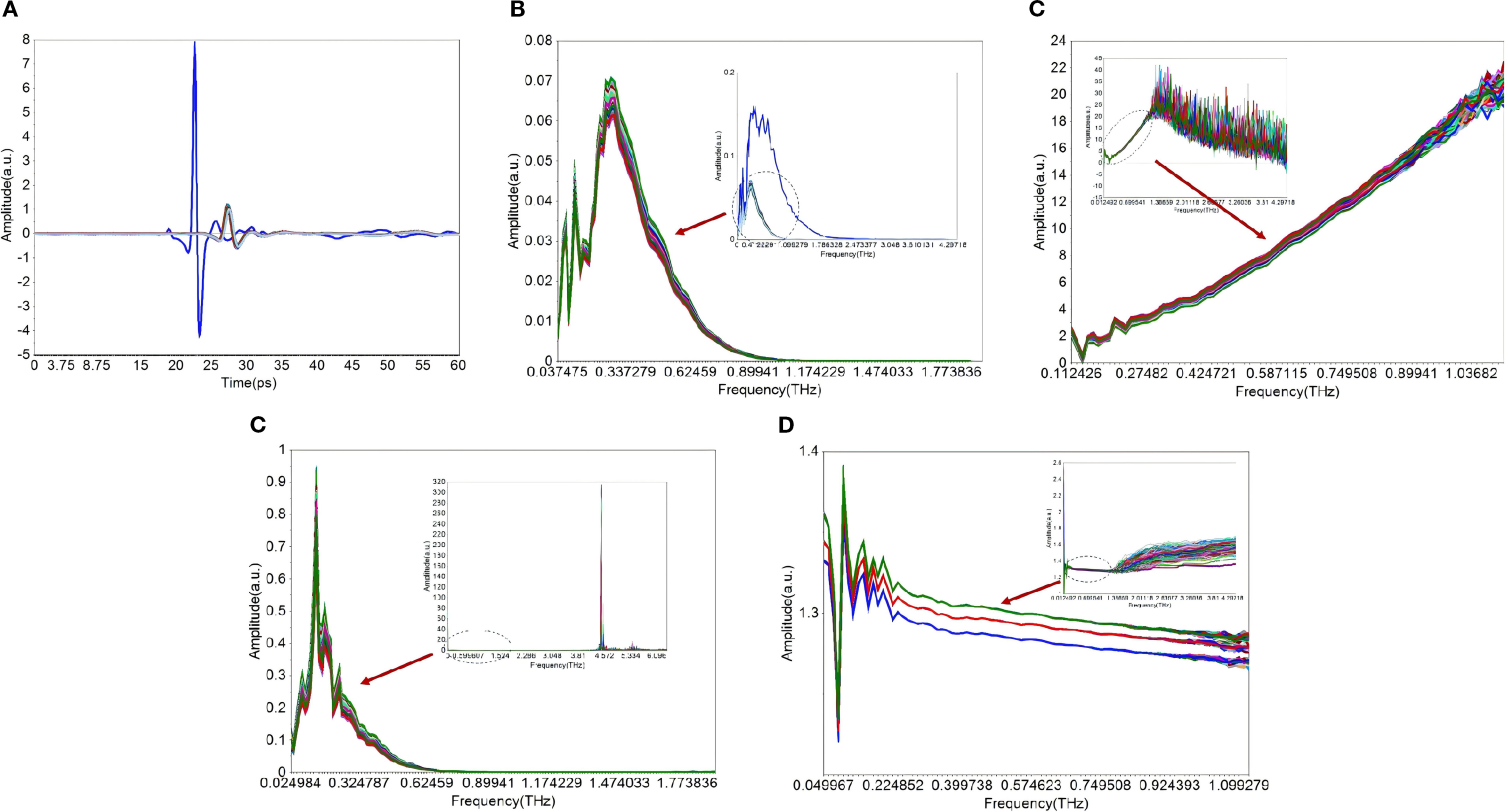
Original and valid THz spectral data of wheat flour samples. **(A)** time-domain; **(B)** frequency-domain; C) absorption coefficient; **(D)** transmittance; **(E)** refractive index. The small picture represents the original spectral data, and the big picture represents the screened valid spectral data.

The **Figure 5A** illustrates the raw THz-TDS signals of wheat flour samples, which exhibited typical pulsed waveform characteristics with the time axis in picoseconds (ps), reflecting the intensity variation of THz radiation passing through the samples with time. The intrinsic optical properties were subsequently extracted through signal transformation and valid data screening. By applying Fourier transformation to the raw THz-TDS signals, a conversion from the time domain to the frequency domain was achieved, yielding frequency-domain signals (0.1-4.5 THz) that align with the spectral range of the THz-TDS system, as depicted in **Figure 5B**. And the **Figures 5C–E** shows that the optical characteristic parameters of the wheat flour samples, including absorption coefficient, transmittance, and refractive index, were determined by analyzing the amplitude and phase changes of the THz signals before and after their passage through the samples and a reference (air), as per the transmission model of terahertz spectroscopy for solid powder samples.

Valid spectral data screening was conducted on the four transformed datasets by setting a signal-to-noise ratio threshold. This process excluded the low-frequency band with severe signal noise and the high-frequency band with significant signal attenuation. Only spectral data with stable signal response and high reliability were retained for subsequent modeling. As shown in **Figure 5**, the valid frequency ranges of the four datasets were narrower compared to the original full spectral range (0.1-4.5 THz). The signal intensity within the valid range exhibited stable and continuous variation, effectively avoiding the interference of invalid spectral information on model construction.

**Figure 6:**
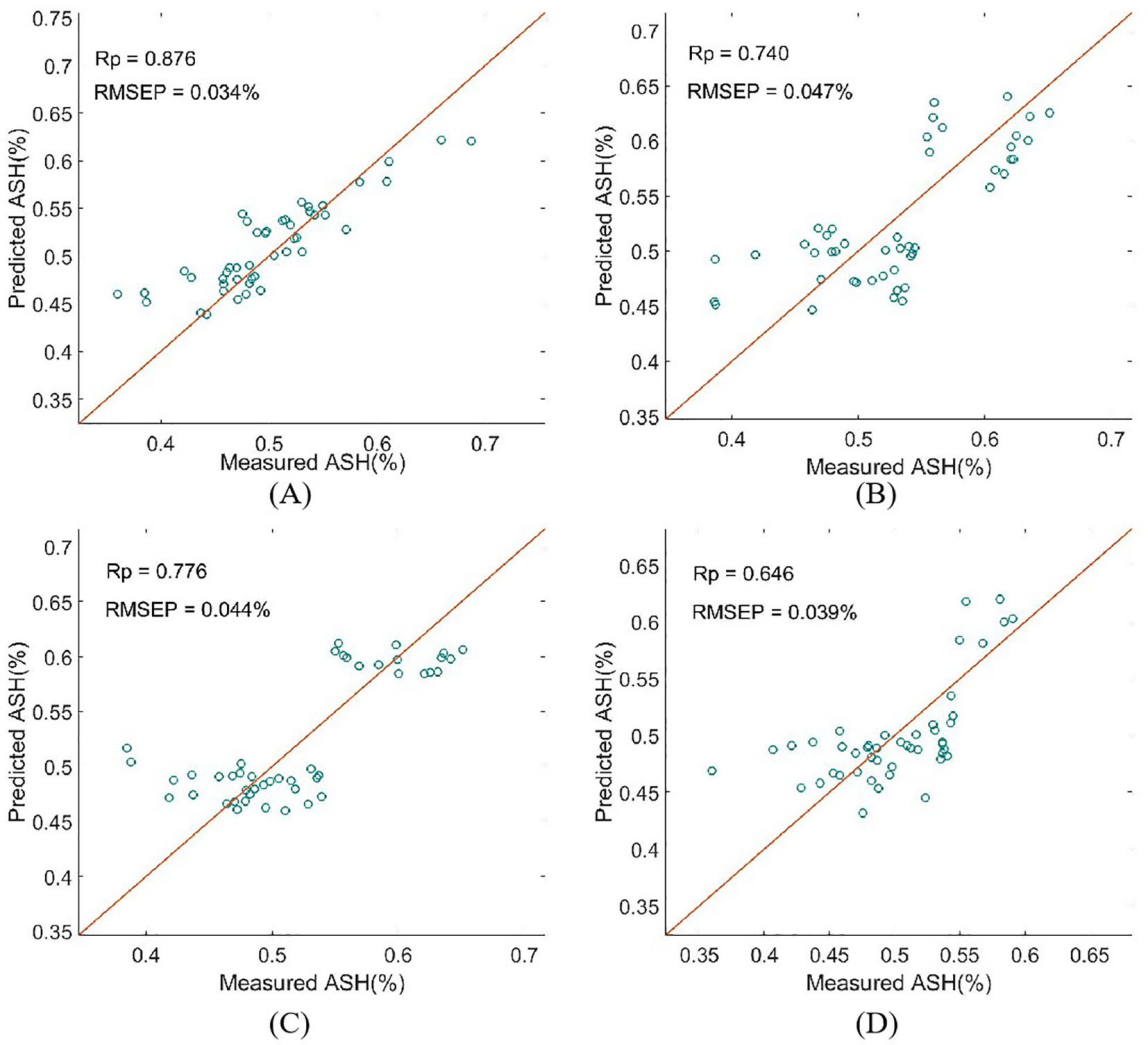
The prediction performance for ash content in wheat flour based on different THz spectral datasets. **(A)** absorption coefficient; **(B)** frequency-domain; **(C)** transmittance; **(D)** refractive index.

The PLSR models for detecting ash content in wheat flour were established using the valid data from frequency-domain signals, absorption coefficient, transmittance, and refractive index. The number of latent variables (LVs) for each model was optimized through 10-fold cross-validation. As shown in **Table 2** and **Figure 6**, the PLSR model based on absorption coefficient demonstrated the best overall performance for ash content detection. It achieved the highest Rc=0.823, Rp=0.876 and RPD = 1.359, and the lowest RMSECV (0.039%), RMSEP (0.034%) and Bias (0.031%). Notably, this model required only 2 LVs to achieve optimal fitting and prediction, indicating a strong intrinsic correlation between absorption coefficient and ash content, and the spectral information was highly efficient for quantitative modeling. In contrast, the models based on frequency-domain signals and refractive index showed relatively poor performance, indicating significant information redundancy and weak correlation with ash content. The transmittance model exhibited moderate performance, with better fitting and prediction effects than the frequency-domain signal and refractive index models, but still inferior to the absorption coefficient model in all evaluation metrics.

**Table 2:** PLSR model performance for ash content detection based on different THz spectral datasets.

Spectral dataset	LVs	Rc	RMSECV (%)	Rp	RMSEP (%)	RPD	Bias (%)
Absorption coefficient	2	0.823	0.039	0.876	0.034	1.359	0.031
Frequency-domain	4	0.696	0.050	0.740	0.047	1.262	0.046
Transmittance	5	0.744	0.045	0.776	0.044	1.313	0.053
Refractive index	12	0.739	0.050	0.646	0.039	1.226	0.034

The superior performance of the absorption model for ash content detection can be attributed to the intrinsic relationship between the absorption coefficient and the chemical composition of wheat flour. Ash content in wheat flour consists of inorganic mineral elements (Ca, Mg, P, K, Fe, etc.), which exhibit unique THz spectral absorption characteristics in the 0.1-4.5 THz range. The absorption coefficient directly reflects the absorption capacity of wheat flour samples for THz radiation at different frequencies, and can sensitively capture the subtle spectral changes caused by variations in mineral element content (ash content). Additionally, absorption coefficient is a normalized optical parameter that eliminates the interference of sample thickness and measurement distance on spectral signals, making it more stable and reliable for quantitative analysis of component content compared to raw frequency-domain signals and transmittance. The valid absorption coefficient data were used as the research object for subsequent spectral preprocessing, characteristic variables extraction, and model optimization in this study.

### 3.3 Effect of spectral data preprocessing

**Figure 7:**
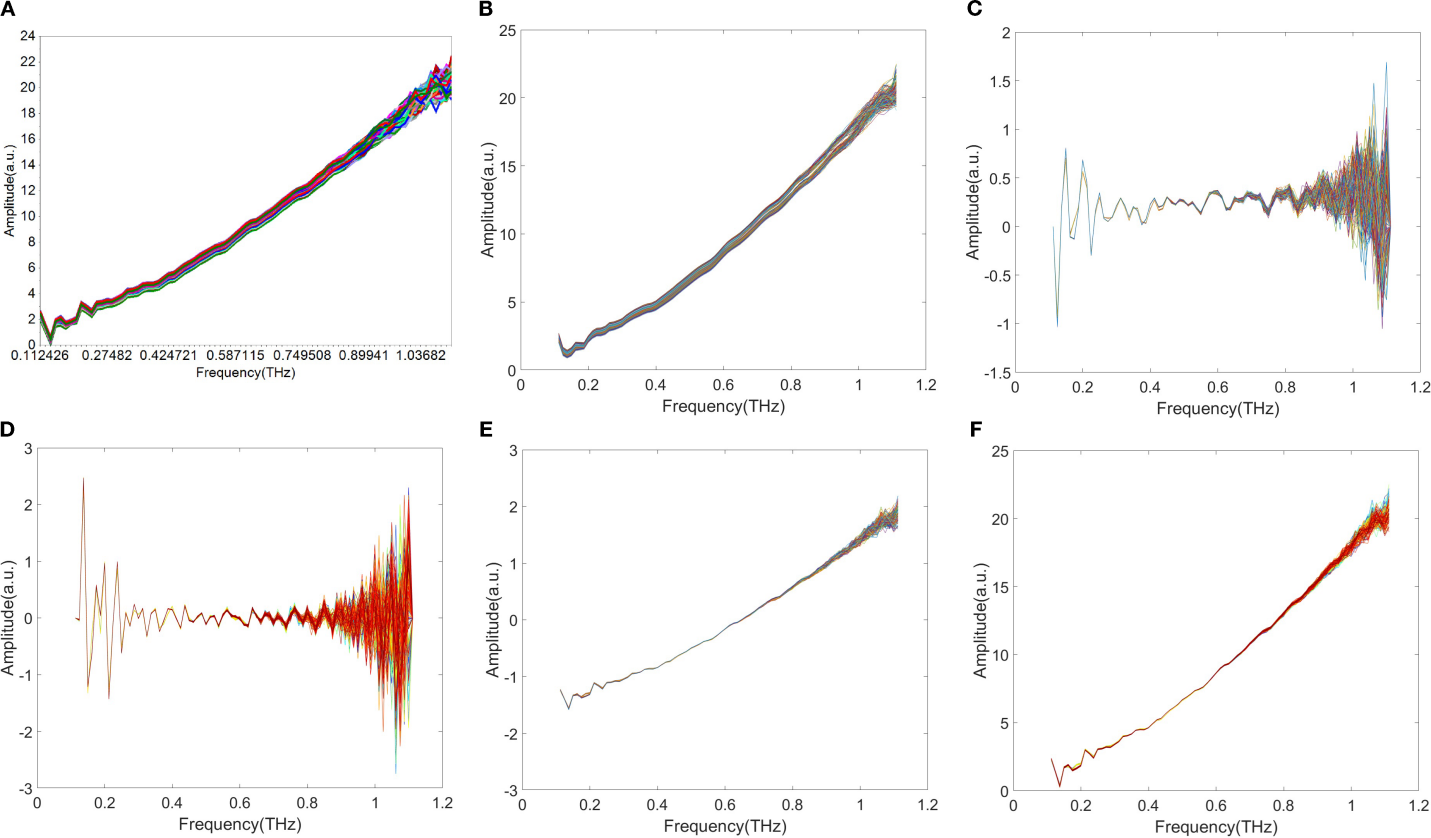
THz absorption coefficient spectra of wheat flour after different preprocessing methods. **(A)** raw; **(B)** SG; **(C)** 1D; **(D)** 2D; **(E)** SNV; **(F)** MSC.

The preprocessing of spectral data was systematically evaluated to enhance the performance of THz absorption coefficient spectra for wheat flour analysis. The **Figure 7** shows that SG, 1D, 2D, SNV, and MSC were applied to suppress noise, correct baseline drift, and eliminate scattering interference. As shown in **Figure 7**, the spectral profiles of SNV and MSC exhibit high visual similarity, which is consistent with the theoretical expectation that both methods address comparable light-scattering effects in Terahertz spectroscopy.

The **Table 3** illustrates that SG smoothing method (3-point average) yielded the best overall performance, with Rc=0.826, RMSECV = 0.040%, Rp=0.850, RMSEP = 0.031%, RPD = 1.486 and Bias=0.030%. This represents a slight improvement in fitting goodness and a clear enhancement in predictive accuracy compared to the raw spectral model. The effectiveness of SG smoothing can be attributed to its ability to effectively suppress high-frequency random noise in the THz absorption signal.

**Table 3:** Performance of PLSR models based on raw and preprocessed THz absorption coefficient spectra.

Preprocessing method	Rc	RMSECV (%)	Rp	RMSEP (%)	RPD	Bias (%)
None	0.823	0.039	0.876	0.034	1.359	0.031
**SG**	**0.826**	**0.040**	**0.850**	**0.031**	**1.486**	**0.030**
1D	0.465	0.064	0.488	0.049	0.458	0.157
2D	0.496	0.063	NAN	NAN	NAN	NAN
SNV	0.560	0.057	NAN	NAN	NAN	NAN
MSC	0.739	0.047	0.688	0.047	0.998	0.174

Bold values indicate that the SG preprocessing method achieved the optimal results.

In contrast, derivative transformations (1D and 2D) significantly degraded the model performance, and the 2D preprocessing even led to invalid prediction outputs (NaN). For the 2D method, the second-order derivative operation significantly amplified high-frequency stochastic noise, particularly in the range above 3.5 THz where the system’s dynamic range begins to decline. This noise amplification resulted in a spectral matrix with a very high condition number, leading to a singular matrix during the PLSR weight calculation $W=XX^{\prime }U{\left({\text{U}}^{\prime }\text{U}\right)}^{-1}$. This numerical instability prevented the model from converging on a stable solution for the independent prediction set.

Scatter correction methods (SNV and MSC) also failed to improve modeling performance, with SNV resulting in invalid prediction outputs and MSC significantly reducing both calibration and prediction correlation coefficients. For the SNV method, the failure originated from the scaling process. Because SNV divides each spectrum by its standard deviation, spectra with regions of near-zero variance—common in the noise-dominated high-frequency bands of THz-TDS—resulted in a division-by-zero error. This generated non-finite values in the feature matrix, rendering the model mathematically invalid for external validation. This suggests that the main interference in the present THz absorption spectra is random noise rather than physical scattering caused by particle size or sample compaction heterogeneity. Therefore, the SG-preprocessed absorption coefficient spectra were adopted for subsequent feature wavelength selection and quantitative model optimization.

### 3.4 Effect of characteristic spectral data extraction

To further reduce spectral dimensionality, eliminate redundant variables, and enhance the correlation between THz absorption signals and ash content, SPA, CARS, GA, and BOSS were applied to the SG-smoothed THz absorption coefficient spectra. These methods aimed to identify the most effective wavelengths for predicting the ash content in wheat flour.

**Figure 8:**
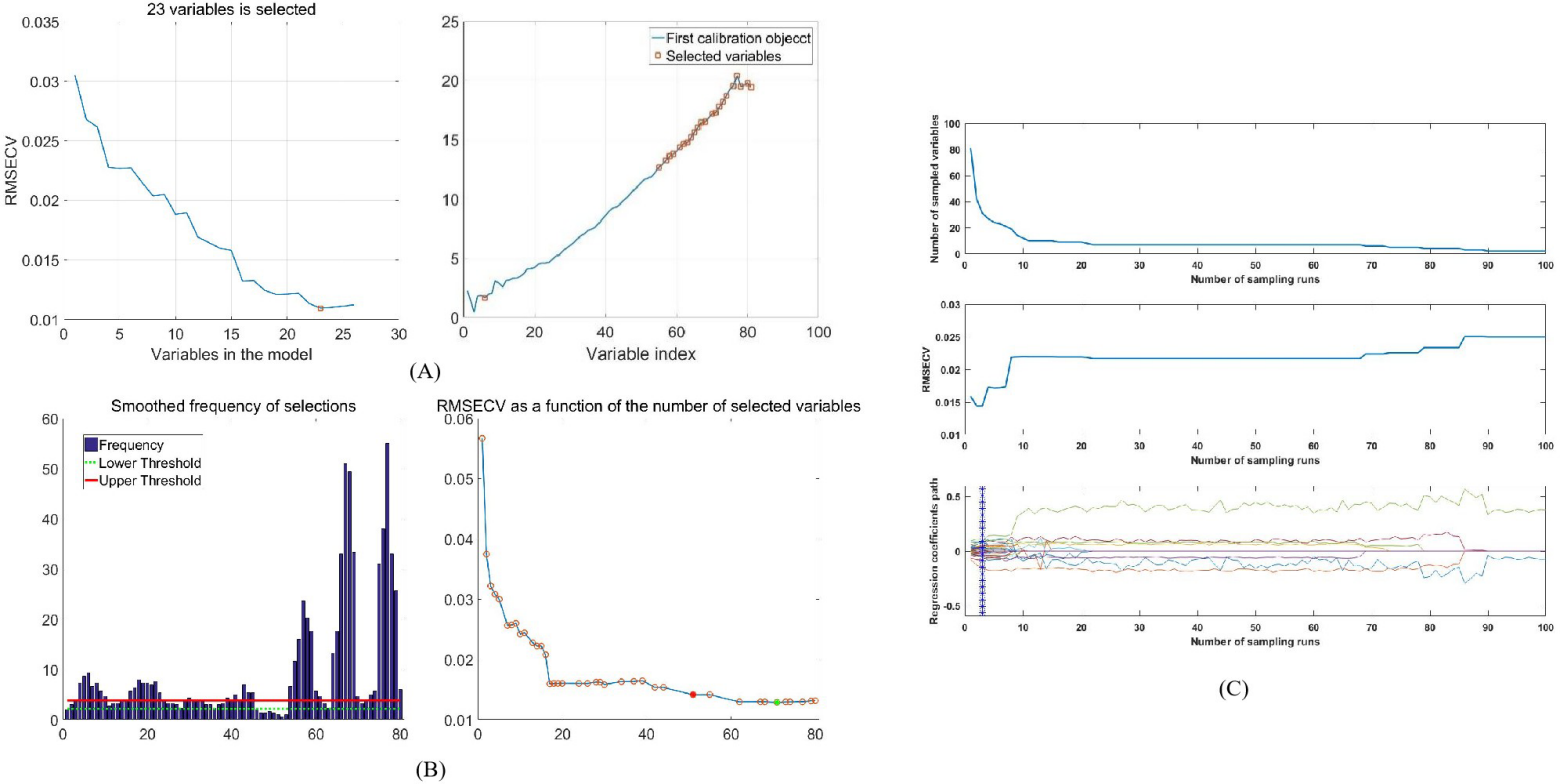
Process of selecting characteristic spectral data by using the **(A)** SPA, **(B)** GA, **(C)** CARS.

**Table 4** summarizes the performance of the PLSR models based on the selected wavelengths using each method. According to **Figure 8A**, SPA identified a subset of 23 characteristic variables that effectively minimized the internal cross-validation error during the selection process. The SPA-PLSR model utilized the fewest variables but showed relatively low performance. With 10 latent variables (LVs), it yielded Rp, RMSEP, RPD and Bias values of 0.934, 0.026%, 2.262 and 0.016%, respectively. **Figure 8B** illustrates the GA wavelength selection process, only variables falling between the established lower threshold (green dashed line) and upper threshold (red solid line) were retained as characteristic variables for the final modeling. GA prioritized 39 variables that demonstrated the highest contribution to reducing model complexity and error. While GA exhibited moderate performance, it suffered from unstable convergence. The GA-PLSR model used 8 LVs, achieving Rp, RMSEP, RPD and Bias values of 0.946, 0.021%, 2.834, and 0.005%, respectively (**Table 4**). For the BOSS algorithm, the maximum number of LVs was set to 20 with 2000 iterations. Performance was assessed via 10-fold cross-validation. BOSS retained 54 effective wavelengths after eliminating redundant variables. The model used 16 LVs and outperformed both SPA and GA. It achieved an Rp of 0.975, an RMSEP of 0.0134%, an RPD of 4.412 and an Bias of 0.004% (**Table 4**).

**Table 4:** Performance of PLSR models based on characteristic wavelengths selected by different algorithms.

Extraction method	Number of variables	LVs	Rc	RMSECV (%)	Rp	RMSEP (%)	RPD	Bias (%)
SPA	23	10	0.882	0.033	0.934	0.026	2.262	0.016
**CARS**	**31**	**11**	**0.977**	**0.015**	**0.976**	**0.011**	**4.804**	**0.004**
GA	39	8	0.933	0.025	0.946	0.021	2.834	0.005
BOSS	54	16	0.979	0.014	0.975	0.013	4.412	0.004

Bold values indicate that the CARS method yielded the best performance.

In the CARS method, Monte Carlo sampling runs were set to 100 with a maximal principal component number of 10. The optimal variable subset was determined by 10-fold cross-validation. **Figure 8C** depicts trends in the number of sampled variables, RMSECV values, and regression coefficient paths. Based on the minimum RMSECV in the 3rd sampling run, CARS selected 31 variables. The CARS-PLSR model used 11 LVs, achieving high Rc of 0.977, Rp of 0.976 and RPD of 4.804, along with low RMSECV of 0.015%, RMSEP of 0.011% and Bias of 0.004%. The RPD value far exceeds 2.5, indicating excellent practical prediction performance. The Bias close to 0 confirms the absence of obvious systematic deviation.

**Figure 9:**
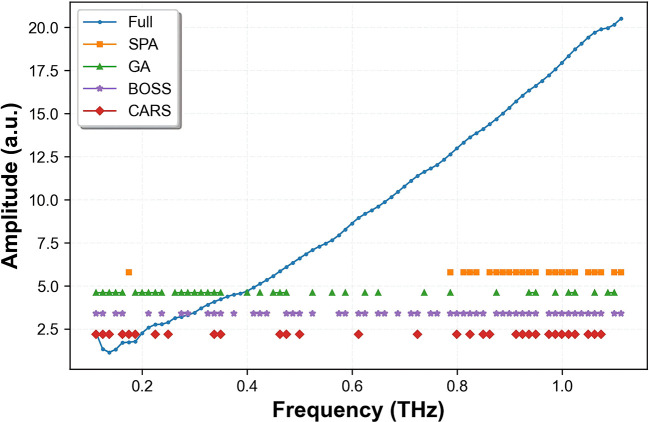
Distribution of characteristic spectral data selected by SPA, GA, BOSS, and CARS from SG-smoothed THz absorption coefficient spectra.

**Figure 9** displays the distribution of wavelengths selected by CARS for the prediction of ash content across the full spectrum. The selected frequencies were 0.112426, 0.124918, 0.13741, 0.162393, 0.174885, 0.187377, 0.224852, 0.249836, 0.337279, 0.34977, 0.462197, 0.474688, 0.499672, 0.612098, 0.724525, 0.799475, 0.824459, 0.849443, 0.861934, 0.911902, 0.924393, 0.936885, 0.949377, 0.974361, 0.986852, 0.999344, 1.011836, 1.024328, 1.049311, 1.061803 and 1.074295 THz. Most of the selected wavelengths were associated with chemical properties. Notably, most of these selected frequencies correspond to the characteristic THz absorption coefficient of inorganic minerals, the main constituents of ash. These include metal cations (e.g., K^+^, Na^+^, Ca^2+^, Mg^2+^) and inorganic salts (e.g., phosphates, carbonates, and oxides). In the low to medium THz region (0.1-1.1 THz), these characteristic frequencies mainly reflect the collective vibration, phonon resonance, and dipole rotation behaviors of inorganic mineral crystals and residual ash components in wheat flour. The strong absorption coefficient responses at these frequencies are highly consistent with the variation of ash content, which explains why the CARS-selected variables significantly improved the prediction accuracy and stability of the quantitative model.

### 3.5 Estimation model for ash content determination

Based on the 31 characteristic wavelengths selected by the SG-CARS strategy, PLSR, MLR, PCR, and SVR regression algorithms were employed to construct quantitative models for ash content in wheat flour.

**Figure 10:**
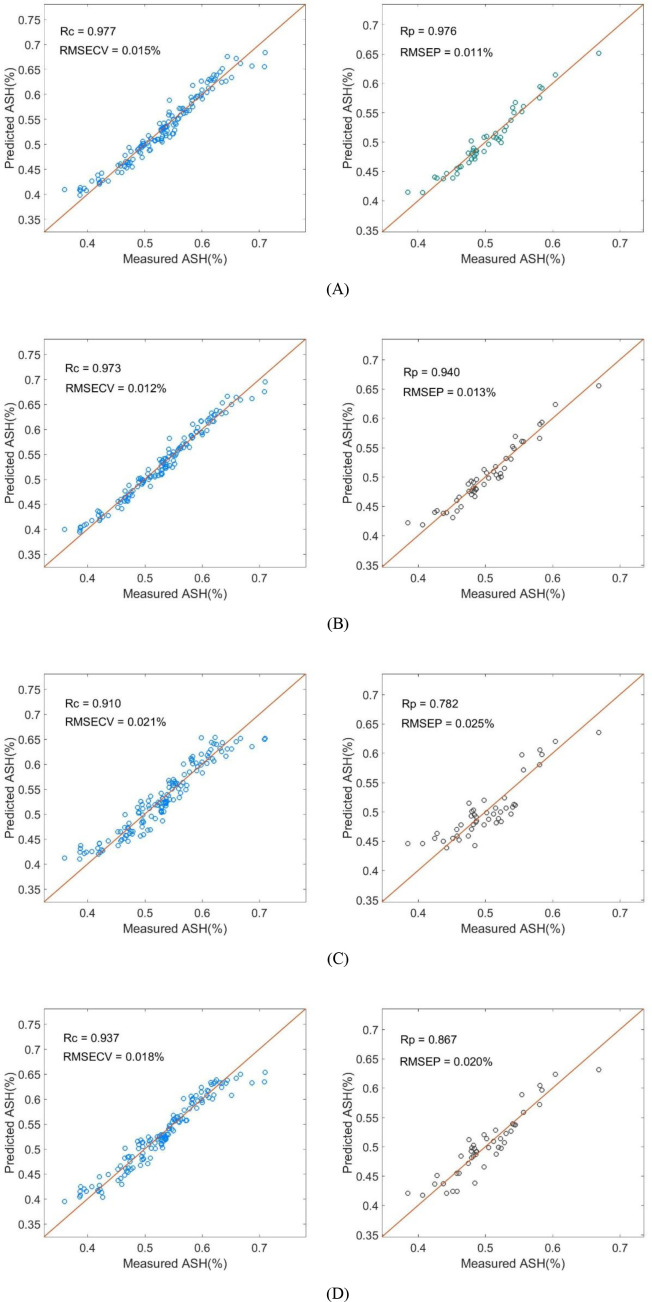
The performance of different predicted model for ash content determination in wheat flour. **(A)** PLSR model, **(B)** MLR model, **(C)** PCR model and **(D)** SVR model.

As shown in **Table 5**, the RPD values for the MLR, PLSR, and SVR models all exceeded 2.5, confirming their excellent predictive capacity for ash content determination. The low Bias values across all models demonstrate that the proposed THz-TDS methodology is free from significant systematic errors. **Figure 10** showed the performance of different predicted model for ash content determination in wheat flour. The SG-CARS-PLSR model achieved the optimal comprehensive performance. It yielded the higher Rc = 0.977, Rp = 0.976 and RPD = 4.804, and the lower RMSECV = 0.015%, RMSEP = 0.011% and Bias=0.004%. The superiority of PLSR lies in its ability to process high-dimensional data while mitigating multicollinearity. Unlike MLR, which fails to address variable redundancy, or PCR, which extracts components based solely on spectral variance without considering the target variable, PLSR simultaneously decomposes the spectral and concentration matrices. This approach maximizes the covariance between spectral features and ash content. Consequently, PLSR retained critical chemical fingerprint information and demonstrated superior generalization capability.

**Table 5:** Performance of quantitative regression models for wheat flour ash content based on SG-CARS selected characteristic wavelengths.

Modeling algorithm	Rc	RMSECV (%)	Rp	RMSEP (%)	RPD	Bias (%)
**PLSR**	**0.977**	**0.015**	**0.976**	**0.011**	**4.804**	**0.004**
MLR	0.973	0.012	0.940	0.013	4.082	0.003
PCR	0.910	0.021	0.782	0.025	2.037	0.008
SVR	0.937	0.018	0.867	0.020	2.679	0.004

Bold values indicate that the PLSR method yielded the best performance.

In contrast, the SG-CARS-MLR model showed reduced prediction stability (Rp=0.940, RMSEP = 0.013%, RPD = 4.082, and Bias=0.003%), likely due to unhandled multicollinearity among variables. The SG-CARS-PCR model performed the poorest among the linear models (Rp=0.782, RMSEP = 0.025%, RPD = 2.037, and Bias=0.008%). Its dimensionality reduction logic ignored the quantitative link between spectra and ash content, thereby retaining variance unrelated to the target component. Similarly, the nonlinear SVR model (Rp=0.867, RMSEP = 0.020%, RPD = 2.679, and Bias=0.004%) failed to match the performance of the linear models. Its reliance on nonlinear mapping assumptions contradicted the observed linear spectral-component correlation. Forcing a nonlinear fit on a linearly correlated dataset likely induced overfitting, compromising the model’s prediction accuracy.

The SG-CARS-PLSR results confirmed CARS as an effective wavelength selection method, which effectively identifies characteristic spectral data associated with inorganic minerals while eliminating redundant noise. **Figure 10A** shows the scatter plot of measured versus predicted ash content for the calibration and prediction sets. The performance gap between linear and nonlinear models underscores the importance of matching modeling assumptions with data characteristics. Therefore, the SG-CARS-PLSR model is determined to be the optimal strategy for ash content determination.

Despite the high performance of the SG-CARS-PLSR model, it is important to acknowledge that other techniques, such as Laser-Induced Breakdown Spectroscopy (LIBS), have demonstrated superior accuracy (R^2^ = 0.992) across a more extensive ash content range (0.48%–1.39%) (Bilge et al., 2016). The current study’s focus on a narrower range (0.36%–0.71%) was designed to optimize sensitivity for high-grade commercial flours. However, we recognize that the limited sample size and range may affect the model’s generalizability across all flour types. Future work will focus on expanding the dataset to include a wider variety of wheat cultivars and a broader distribution of ash contents, specifically incorporating whole wheat and industrial-grade flours. This expansion will be critical to further validate the robustness of THz-TDS and enhance its persuasiveness as a universal tool for the milling industry.

## 4 Conclusions

This study established a rapid, nondestructive, and high-precision method for determining ash content in wheat flour by integrating terahertz time-domain spectroscopy (THz-TDS) with optimized chemometric strategies. A total of 183 wheat flour samples from three major cultivars in southwest China were analyzed, with reference values obtained via standard high-temperature incineration. The results demonstrated that the THz absorption coefficient exhibited the strongest correlation with ash content. Savitzky–Golay (SG) smoothing was identified as the optimal preprocessing method, effectively suppressing noise while preserving critical spectral features related to inorganic mineral components. CARS successfully screened 31 effective wavelengths (0.11–1.07 THz) closely associated with ash constituents, significantly reducing spectral dimensionality and model complexity. Comparative modeling confirmed PLSR outperformed MLR, PCR and SVR, reflecting the dominant linear relationship between THz absorption and ash content. The final optimized SG-CARS-PLSR model achieved excellent performance metrics, with Rc​=0.977, RMSECV = 0.015%, Rp​=0.976, RMSEP = 0.011%, RPD = 4.804, and Bias=0.004%. This proposed strategy provides a reliable, reagent-free, and efficient tool for rapid wheat flour quality evaluation. The successful identification of characteristic wavelengths (0.1–4.5 THz) effectively reduced model complexity while maintaining high sensitivity to inorganic mineral variations. Although this study focuses on a specific ash content range (0.36%–0.71%) common in high-grade flours, future research will aim to expand the sample size and detection range to further enhance the model’s universal applicability. Overall, THz-TDS proves to be a robust and efficient alternative to traditional chemical methods, offering substantial potential for real-time quality control and process optimization in the modern milling industry.

## Data Availability

The original contributions presented in the study are included in the article/supplementary material. Further inquiries can be directed to the corresponding authors.
